# Vascular characteristics and haemodynamics of severe and mild pterygium: a quantitative analysis

**DOI:** 10.1080/07853890.2025.2580009

**Published:** 2025-10-28

**Authors:** Jiaxin Han, Qianwen Gong, Xingwei Zhu, Ruiting Ji, Meng Li, He Wang, Jia Qu, Liang Hu

**Affiliations:** ^a^ National Clinical Research Center for Ocular Diseases, Eye Hospital, Wenzhou Medical University, Wenzhou, China; ^b^ State Key Laboratory of Ophthalmology, Optometry and Visual Science, Eye Hospital, Wenzhou Medical University, Wenzhou, China; ^c^ Department of Ophthalmology, The Affiliated Hospital of Xuzhou Medical University, Xuzhou, China

**Keywords:** Pterygium, vascular characteristics, haemodynamics, OCTA, FSLB

## Abstract

**Purpose:**

To investigate vascular characteristics and hemodynamic changes in mild and severe pterygium and explore correlations among clinical parameters.

**Design:**

Cross-sectional observational study.

**Methods:**

Sixty-three patients (68 eyes) with pterygium, classified into mild (32 eyes) and severe (36 eyes) groups, and 30 healthy controls (36 eyes) were included. Pterygium length and area were measured via slit-lamp photography. Optical coherence tomography angiography assessed pterygium thickness and vessel density. A functional slit-lamp biomicroscopy measured vessel diameter, vessel length and blood flow velocities. Correlation analysis was performed for each parameter.

**Results:**

The vessel density was significantly higher in the pterygium groups compared to the control group (*p* < 0.001). Moreover, the vessel density was significantly greater in the severe pterygium group than in the mild pterygium group (*p* = 0.002). Vessel diameter and length varied significantly (*p* = 0.01, *p* = 0.003), with the mild group showing the largest diameter and the severe group the shortest length. No significant differences in hemodynamic parameters were found. Pterygium length and area were positively correlated with age (*p* < 0.001). The area under the curve (AUC) for pterygium thickness in differentiating mild from severe pterygium was 0.79.

**Conclusions:**

Pterygium demonstrates elevated ocular surface vascular density that correlates with disease severity. Changes in vascular morphology include vessel dilation and increased branching, with dilation most prominent in mild pterygium and branching in severe pterygium. These findings enhance the understanding of pterygium progression and may inform severity-based clinical management.

## Introduction

Pterygium is a common ocular surface disorder characterized by wing-shaped, fibrovascular proliferation that extends from the bulbar conjunctiva onto the cornea [1]. It is a prevalent ocular surface disease, with an estimated 108.65 million cases reported in China alone  [2]. The prevalence of pterygium varies significantly depending on the study population. Globally, the prevalence of pterygium is about 12%; however, it is as high as 58.8% in the Brazilian Amazon [3,4]. Beyond its aesthetic concerns, pterygium can cause ocular discomfort, vision loss, reduced motility, and diplopia. Fernandes et al. [4] reported that pterygium leads to visual impairment in 14.3% and blindness in 3.9% of cases, making it the second most common cause of visual impairment and blindness (10.5%) when considering best-corrected visual acuity. Despite efforts to characterize the pathogenesis of pterygium, the specific mechanisms behind pterygium development and progression remain unclear.

The pathology is marked by fibrovascular tissue hyperplasia, with vascular changes being more prominent than fibrosis [5]. The blood supply for pterygium comes from the conjunctival circulation, and alterations in the bulbar conjunctival vasculature’s morphology and haemodynamics can lead to vascular dysfunction and vasculopathy and subsequent pathophysiological changes [6]. Traditional vascular studies of pterygium have used methods like conjunctival indocyanine green contrast, conjunctival sodium fluorescein contrast and immunohistochemistry [6,7], which are somewhat invasive, unquantifiable and not suitable for large-scale clinical use. Recent advancements in medical imaging technology have introduced non-invasive imaging techniques like functional slit-lamp biomicroscopy (FSLB) and anterior segment optical coherence tomography angiography (AS-OCTA) for evaluating ocular surface vascular conditions. Methods that allow quantitative, serial and non-invasive assessment of pterygium vasculature will undoubtedly provide more information about the pathogenesis and characteristics of pterygium. Zhao et al. [8] found higher vessel density in pterygium compared to normal conjunctiva using AS-OCTA. Still, the differences in vessel density among various pterygium grades remain unclear, and few studies have examined the haemodynamics of pterygium. Understanding the vascular characteristics and hemodynamic changes in different grades of pterygium could enhance our comprehension of the disease’s physiological and pathological processes and guide treatment decisions.

Currently, *in vivo* studies on pterygium vasculature are limited. This study used FSLB and OCTA to explore alterations in clinical parameters like vessel density and haemodynamics in different grades of pterygium and investigate their correlations between the parameters. Through this study, a deeper understanding of the changes in ocular vascular characteristics and clinical parameters, including haemodynamics, in patients with different grades of pterygium is anticipated. The findings are expected to provide valuable insights into the pathogenesis of the disease.

## Methods

### Study population

This prospective cross-sectional study was approved by the Ethics Committee of Wenzhou Medical University (code: 2022-126-K-96-01) and conducted in accordance with the Declaration of Helsinki. Written informed consent was obtained from all participants. The study comprised 63 adult patients (aged 18 years or older) with primary pterygium (68 eyes) and 30 healthy participants (36 eyes) who served as controls, enrolled from April 2022 to October 2023. The control group consisted of age- and sex-matched individuals recruited from outpatient health examinations. Exclusion criteria for both groups encompassed pseudo pterygium, recurrent pterygium, uveitis, glaucoma, ocular tumours, history of corneal contact lenses, ocular surgeries, traumatic eye injury, as well as systemic diseases affecting the ocular surface, such as hypertension and diabetes. Additionally, control subjects were required to have no ocular surface pathology, with the absence of pterygium being the key distinguishing feature from the patient group.

### Pterygium grouping

**Figure 1.**
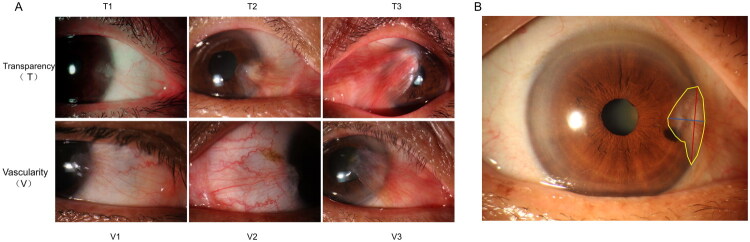
Grading pterygium severity and quantifying lesion size. (A) Representative images of pterygium with transparency grades T1 to T3 and vascularization grades V1 to V3. (B) Methodology of measuring and quantifying pterygium size. Length, width and area of the pterygium were measured using ImageJ software (National Institutes of Health, Bethesda, MD). The two intersection points where the pterygium crossed the corneal limbus were identified, with the distance between these points representing the width of the pterygium (red line). A line was drawn from the outermost end of the pterygium to the corneal limbus, perpendicular to the first line, defining the length of the pterygium (blue line). A quantitative area grading was conducted by carefully delineating the head of the pterygium (yellow line). After delineation, the software automatically calculated the area.

The pterygium was graded independently by two ophthalmologists, each with over 5 years of clinical experience, based on the transparency and vascularity of the pterygium tissue (Q.W.G. and X.W.Z.). In cases of disagreement between the two ophthalmologists, a third senior ophthalmologist graded the pterygium (H.W.). The two graders showed good agreement. Grading criteria proposed by Tan et al. [9] and Kim et al. were utilized to assess pterygium transparency (grades T1–T3) and vascularity (grades V1–V3), respectively (see Table 1 and Figure 1A). This combined grading approach was adopted because pterygium severity is multifactorial, and integrating both transparency and vascularity parameters provides a more comprehensive assessment of disease activity than single-parameter systems. The transparency grade (T) reflects the degree of fibrovascular tissue thickening, whereas the vascularity grade (V) quantifies the level of pathological angiogenesis; these two dimensions are biologically correlated [9,10]. For clinical utility and statistical clarity, pterygia was categorized into mild and severe types. The mild type comprised pterygiums of grade T1 and V1 or V2, whereas the severe type included pterygiums of grade T2 or T3 and V2 or V3 [10].

**Table 1.** Clinical grading system of pterygium.

Grade	Characteristics
Translucency – grade T	
T1	Pterygium with distinguished episcleral vessels underlying its body
T2	Pterygium with partially obscured episcleral vessel details underlying its body
T3	Fleshy pterygium with obscured episcleral vessels underlying its body
Vascularity – grade V	
V1	Minimal vascularization
V2	Moderate vascularization with enlarged vessels
V3	Marked vascularization with engorged vessels

### Experimental design

All subjects underwent slit-lamp microscopy (SLE-8E; KangHuaRuiMing Science Technology Co., Ltd., Chongqing, China), FSLB and AS-OCTA (VG100D; SVision Imaging Co., Ltd., Luoyang, China). These examinations were conducted by the same operator (J.X.H.) in a consistent environment, with the examination room maintained at a temperature of approximately 20–25 °C and humidity of approximately 45–60%.

#### Slit-lamp assessment

All patients underwent slit-lamp microscopy and anterior segment photography. The parameters of the pterygium head, including its width (mm), length (mm) and area (mm^2^), were quantified using ImageJ software (National Institutes of Health, Bethesda, MD). The software was calibrated using a standardized scale bar captured in the same imaging session to ensure measurement accuracy. The quantitative analyses were performed by a single, independent evaluator who was blinded to the clinical grading of the subjects (R.T.J.). To achieve this blinding, all images were assigned random numerical codes prior to analysis, and any identifying clinical information was removed. The fibrous method [11] was employed to delineate the pterygium head, defining it as any fibrovascular tissue crossing the corneal limbus, including any associated corneal clouding (Figure 1B).

#### Anterior segment-optical coherence tomography angiography assessment

All scans were performed by the same physician using a swept-source OCTA device operating near 1050 nm, with a speed of 100,000 A-scans per second and a wide field of 56°. This device integrates optical coherence tomography (OCT) and OCTA for both the anterior and posterior segments. The scanning area was tangent to the corneoscleral margin at the 3 o’clock or 9 o’clock position of the left or right eye, respectively [8]. Scans were captured using the AS Angio scan mode, featuring a scan depth of 4.1 mm. Each 6 × 6 mm^2^ volume scan consisted of 384 A-scans per B-scan across 384 B-scan locations. The OCTA B-scans were meticulously reviewed, and the examiner manually corrected delamination errors. Vessel density, measured by OCTA, was defined as the percentage of vessel area and was measured automatically using the built-in software (Version 1.44.2; SVision Imaging Co., Ltd., Luoyang, China). Pterygium thickness was manually measured at the limbus (horizontal axis is the long axis of the pterygium) using the software’s scale [12]. Both pterygium vessel density and thickness were measured three times, and the average value was used for analysis.

#### Functional slit-lamp biomicroscope assessment

The FSLB system consisted of a slit-lamp microscope integrated with a high-definition digital camera (EOS 60D; Canon Inc., Tokyo, Japan) equipped with an external green filter. The camera’s built-in movie crop function inherently provides an equivalent magnification of approximately ×7, which, when combined with the built-in slit-lamp, provides optical magnification capabilities of up to ×30, resulting in a total magnification of approximately ×210 [13]. FSLB has been utilized in previous studies, and its procedures have been well described [7,14–16].

**Figure 2.**
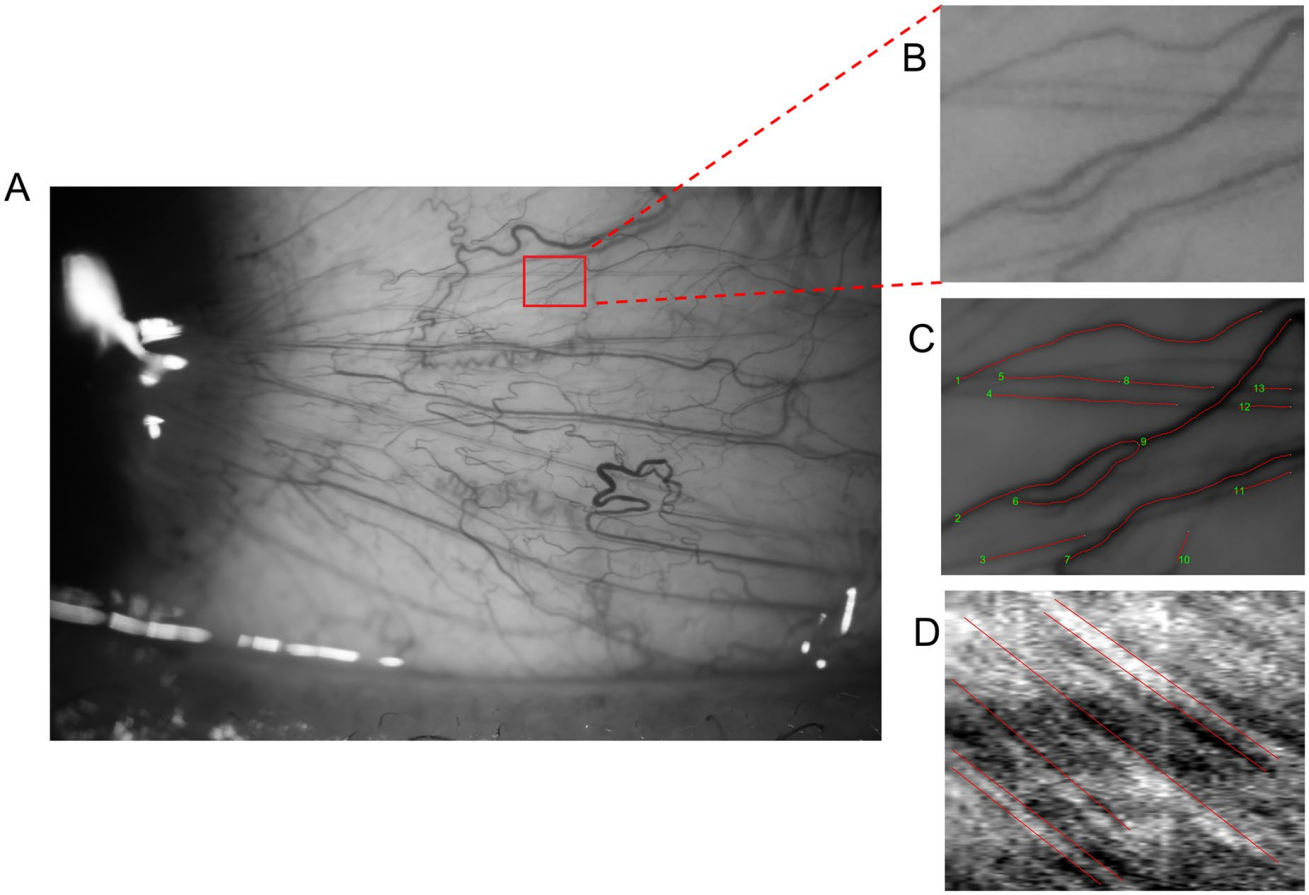
Flowchart of blood flow dynamics video processing. First, functional slit-lamp biomicroscopy captured the video within the region outlined by the red box (A). Subsequently, software such as MATLAB was employed to compensate for eye movements in the recorded video and to convert it into an image (B). The software automatically detected blood vessel signals within the image and delineated the blood vessel contours (C). A red line was manually drawn based on the signals of red blood cell flow, from which relevant parameters were derived (D).

For the dynamic blood flow video, the slit lamp was set to ×25 magnification, with a field of view measuring 1.131 mm × 0.849 mm. The camera was operated in video mode with an ISO 400, a shutter speed of 1/60, and an image size of 640 × 480 pixels, providing a resolution of up to 3.91 µm. Blood flow dynamics videos were captured from six nasal lateral bulbar conjunctiva or pterygium body regions (the red box in Figure 2A illustrates one of the six captured regions). Using custom-developed software, parameters including vessel diameter (*D*, µm), vessel length (*L*, µm), axial blood flow velocity (Va, mm/s), cross-sectional blood flow velocity (Vs, mm/s) and blood flow volume (*Q*, pl/s) [13,15,17].

The process began with cutting and converting the video to the AVI format, followed by compensating for eye movements using MATLAB (Version R2021a; The MathWorks, Inc., Natick, MA) and identifying vascular signals in at least 30 continuous frames. To measure blood flow velocity, a space-time image was generated where the *y*-axis denotes the vessel length, and the *x*-axis signifies the time in the video. The blood flow signal was delineated as a red line segment, and its slope was used to determine Va through the linear equation *y* = *mx* + *b* (Figure 2). Parameter *D* was defined as the full width at half maximum of the intensity profile. Vs and *Q* were calculated using the equations provided by Koutsiaris et al. [18].

### Statistical analysis

The required sample size was determined using the Pearson package in R (Version 4.2.1; R Foundation for Statistical Computing, Vienna, Austria), with a preset effect size of *f* = 0.4, a statistical test power of 1 − beta = 0.8, and a significance level of alpha = 0.05, indicating that at least 22 eyes per group were necessary. To meet the sample size requirements, we included a minimum of 32 eyes per group. Statistical analyses were conducted using SPSS (Version 26.0; IBM Corp., Armonk, NY). The normality of continuous variables was assessed using the Shapiro–Wilk test. Sex differences were assessed using the Chi-square test. Continuous variables were expressed as mean ± standard deviation (SD). When the data satisfied normal distribution and Chi-square test assumptions, a *t*-test was used for comparisons between two groups, one-way ANOVA was used for comparisons among three groups, and two-by-two comparisons between the groups were performed using the least significant difference test. Nonparametric tests were applied when normality or Chi-square test assumptions were not met. The Mann–Whitney *U*-test was used for comparisons between two groups. For comparisons among three groups, the Kruskal–Wallis test was employed, followed by post hoc pairwise comparisons with Bonferroni’s correction. Statistical significance was set at *p* < 0.05. For correlation analyses, Pearson’s correlation was used when both variables were continuously and normally distributed. Spearman’s correlation was used if at least one variable was not normally distributed. When one variable was binary and the other continuous, a point-biserial correlation was performed. Receiver operating characteristic (ROC) curves were analysed to evaluate the accuracy of clinical parameters for diagnosing different grades of pterygium, with area under the curve (AUC) values and 95% confidence intervals (95% CIs) calculated. All tests were two-tailed, with significance set at *p* < 0.05.

## Results

### Baseline characteristics and morphological parameters

In this study, there were no statistically significant differences in age (*p* = 0.768) and sex (*p* = 0.907) among the mild pterygium group (32 individuals; 32 eyes; 14 males/18 females), the severe pterygium group (34 individuals; 36 eyes; 14 males/20 females) and the control group (30 individuals; 36 eyes; 14 males/16 females) (Table 2). The mean lengths of the mild pterygium and severe pterygium groups were 3.10 ± 1.20 mm (range: 0.98–5.01 mm) and 3.91 ± 1.31 mm (range: 1.09–6.60 mm), respectively, with the differences being statistically significant (*t* = −2.62, *p* = 0.011). The mean areas of the pterygium in the mild and severe groups were 6.24 ± 1.10 mm^2^ (range: 1.86–29.13 mm^2^) and 9.74 ± 1.62 mm^2^ (range: 4.90–42.08 mm^2^), respectively, with a statistically significant difference (*p* = 0.018). Additionally, the thickness of the pterygium was significantly greater in the severe group compared to the mild group (*t* = −4.85, *p* < 0.001) (Table 3).

**Table 2.** Basic information of research subjects in each group.

Group	Number	Sex ratio (male/female, *n*/*n*)	Age (mean ± SD)
Mild pterygium group	32	14/18	61.30 ± 10.00
Severe pterygium group	34	14/20	62.03 ± 9.43
Control group	30	14/16	63.00 ± 8.77
*χ*^2^/*F*		0.195	0.276
*p*		0.907	0.768

Sex differences were assessed using the Chi-square test (*χ*^2^); age differences were assessed using one-way analysis of variance (*F*).

**Table 3.** Clinical parameters of pterygium and control group.

	Control group	Mild pterygium group	Severe pterygium group	*p*
Length of pterygium (mm)	–	3.10 ± 1.20	3.91 ± 1.31	0.011*
Area of pterygium (mm^2^)	–	6.24 ± 1.10	9.74 ± 1.62	0.018*
Thickness of pterygium (µm)	–	403.72 ± 105.26	518.21 ± 89.58	<0.001*
Vessel density (%)	53.26 ± 7.32	63.16 ± 9.37	69.59 ± 8.61	<0.001*
*D* (µm)	16.64 ± 1.56	17.96 ± 2.12	16.99 ± 1.73	0.01*
*L* (µm)	163.46 ± 20.69	167.50 ± 23.14	150.35 ± 20.03	0.007*
Va (mm/s)	0.63 ± 0.21	0.65 ± 0.32	0.72 ± 0.36	0.814
Vs (mm/s)	0.45 ± 0.15	0.46 ± 0.22	0.51 ± 0.25	0.729
*Q* (pl/s)	143.21 ± 60.93	159.00 ± 87.53	157.96 ± 106.22	0.860

*D*: vessel diameter; *L*: vessel length; Va: axial blood flow velocity; Vs: cross-sectional blood flow velocity; *Q*: blood flow volume.

Data are presented as mean ± SD. *p* Values listed in the table are for comparisons among the groups (control, mild and severe) and not pairwise analyses. Nonparametric tests were applied to area of pterygium, *L*, Va, Vs and *Q*; length of pterygium and thickness of pterygium were analysed using *t*-tests; vessel density and *D* were evaluated by one-way ANOVA.

* Statistically significant differences (*p* < 0.05).

### Vascular parameters

#### Vessel density

**Figure 3.**
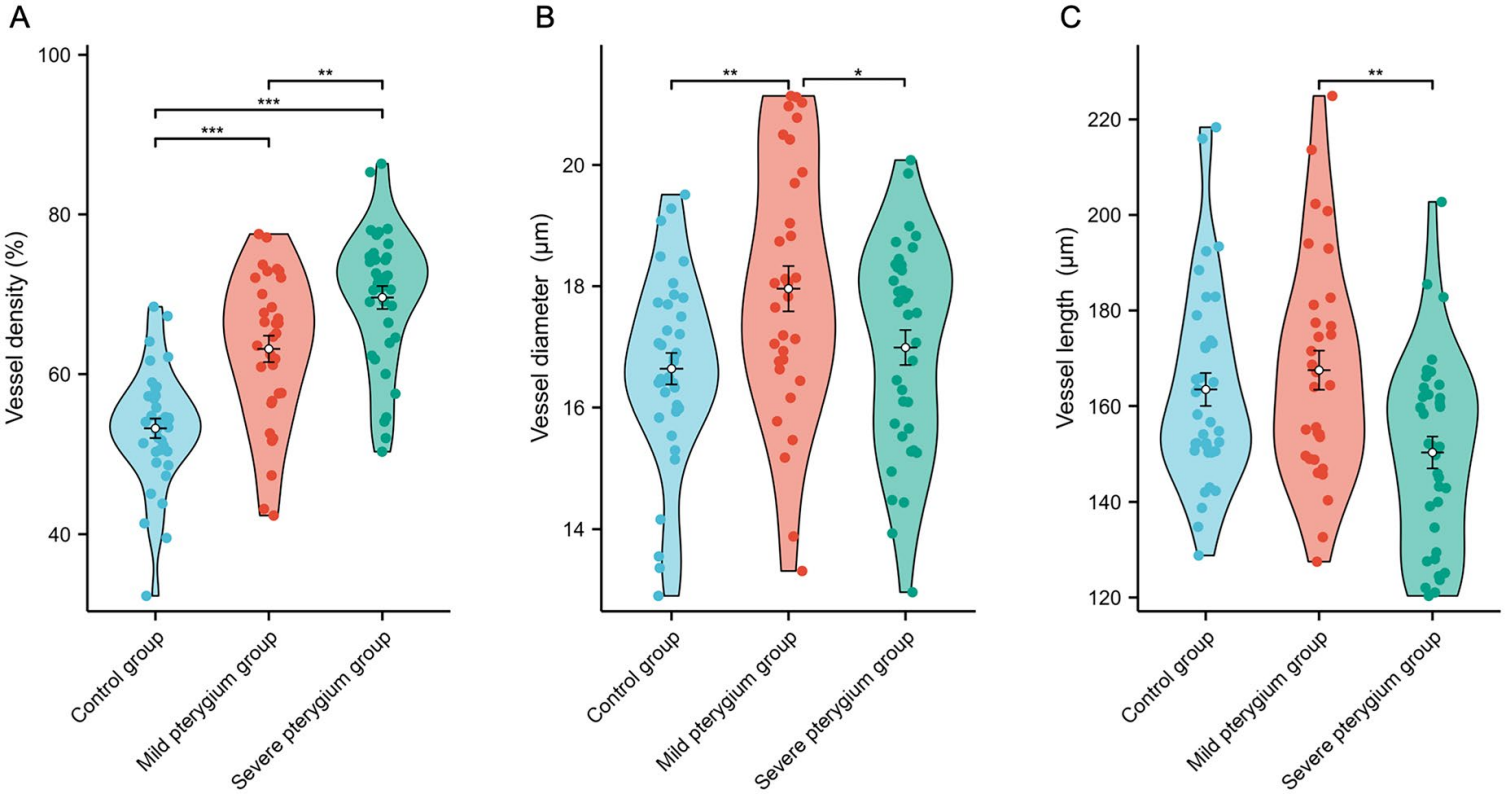
Violin plots illustrating the distribution of vascular parameters among the control group, mild pterygium group and severe pterygium group. (A) Vessel density (%); (B) vessel diameter (μm); (C) vessel length (μm). Statistical significance: *p < 0.05, **p < 0.01 and ***p < 0.001.

The vessel density increased progressively across the control, mild pterygium and severe pterygium groups, with values of 53.26 ± 7.32%, 63.16 ± 9.37% and 69.59 ± 8.61%, respectively (Table 3). The differences in the vessel density among the three groups were statistically significant (*F* = 34.14, *p* < 0.001). Post hoc pairwise comparisons showed significant differences between: the mild group and control group (*p* < 0.001), the severe group and control group (*p* < 0.001), and the mild and severe groups (*p* = 0.002) (Figure 3A).

#### Vessel morphometry

The difference in vessel diameter among the three groups was statistically significant (*F* = 4.80, *p* = 0.01). Specifically, post hoc comparisons demonstrated statistically significant differences between the mild pterygium group and the control group (*p* = 0.003), as well as between the mild and severe pterygium groups (*p* = 0.029). However, no significant difference was observed between the severe pterygium group and the control group (*p* = 0.413) (Figure 3B). Similarly, vessel length differences among the three groups were also statistically significant (*H* = 10.04, *p* = 0.007). Post hoc pairwise comparisons indicated that the difference in vessel length reached statistical significance only between the mild and severe pterygium groups (*p* = 0.009), while no significant differences were detected in any other group comparisons (all *p* > 0.05) (Figure 3C). The highest values for vessel diameter and length were observed in the mild pterygium group: 17.96 ± 2.12 µm and 167.50 ± 23.14 µm, respectively.

#### Hemodynamic trends

No statistically significant differences were found in Va, Vs or *Q* among the three groups (*p* > 0.05). However, blood flow velocities (Va and Vs) showed an increasing trend from the control group to the mild and severe pterygium groups (Va: 0.63 ± 0.21 mm/s, 0.65 ± 0.32 mm/s and 0.72 ± 0.36 mm/s, respectively; Vs: 0.45 ± 0.15 mm/s, 0.46 ± 0.22 mm/s and 0.51 ± 0.25 mm/s) (Table 3).

### Correlates of disease severity

**Figure 4.**
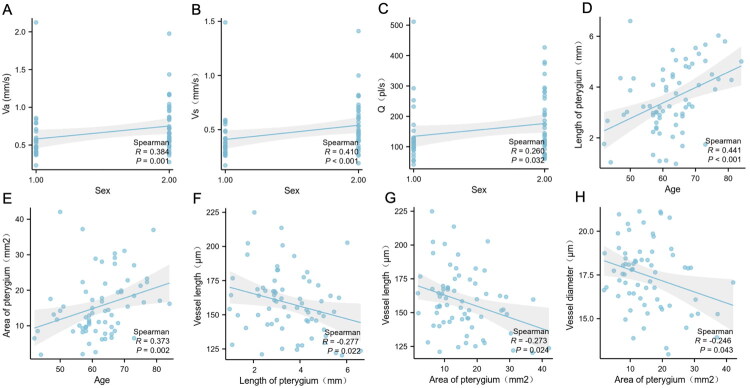
Correlation analysis of clinical parameters. Sex: 1 = male; 2 = female; Va: axial blood flow velocity; Vs: cross-sectional blood flow velocity; Q: blood flow volume.

Correlation analysis between parameters in the mild and severe pterygium groups (Figure 4) revealed that the female sex was positively correlated with Va (*r* = 0.405, *p* < 0.001), Vs (*r* = 0.410, *p* < 0.001) and *Q* (*r* = 0.260, *p* < 0.05). Additionally, both pterygium length (*r* = 0.441, *p* < 0.001) and area (*r* = 0.373, *p* < 0.001) showed significant positive correlations with age. The length of blood vessels was weakly negatively correlated with pterygium length (*r* = −0.277, *p* < 0.05) and area (*r* = −0.273, *p* < 0.05). Furthermore, a weak negative correlation was observed between vessel diameter and pterygium area (*r* = −0.246, *p* < 0.05).

### Diagnostic performance of biomarkers

**Figure 5.**
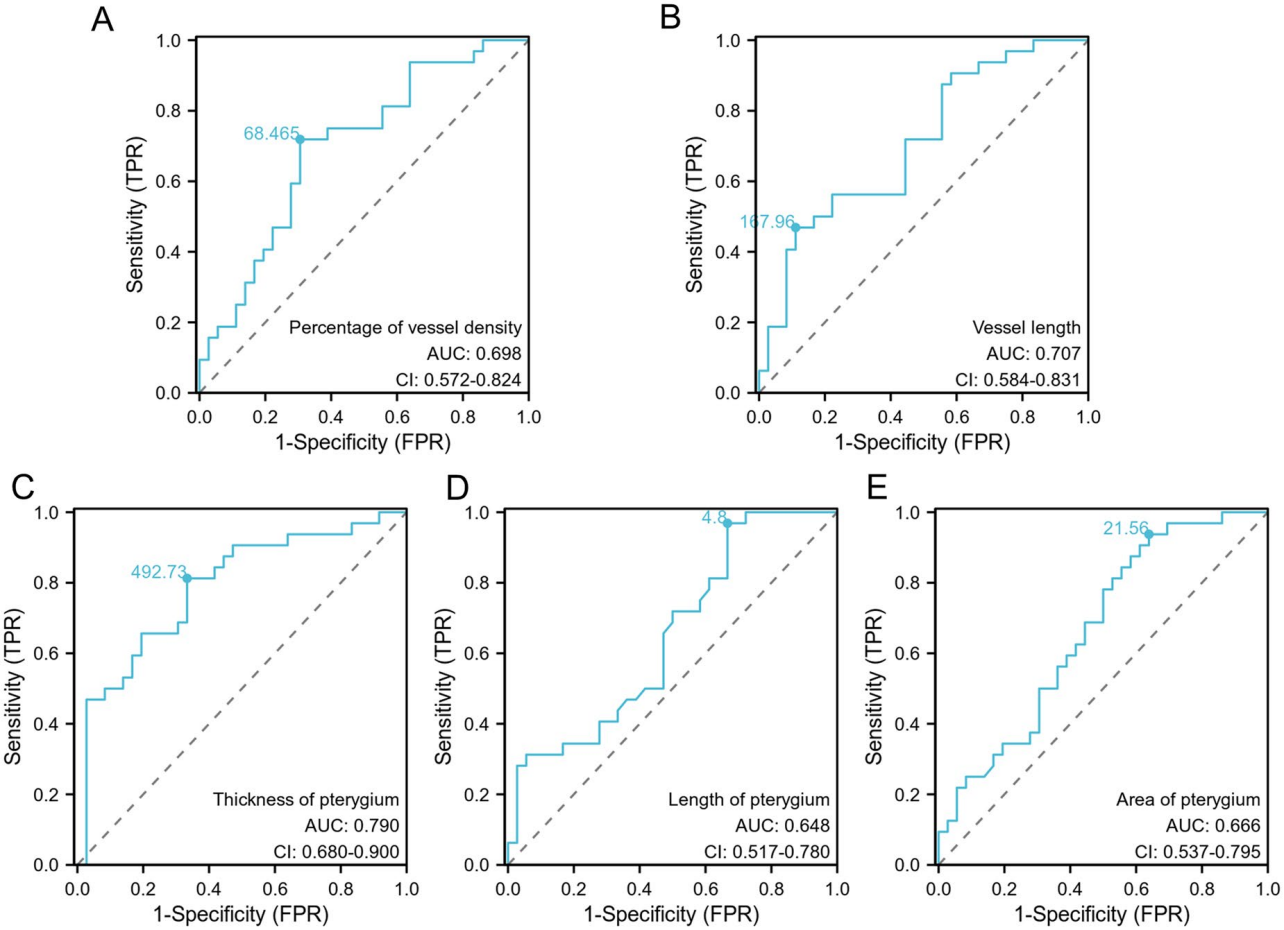
Receiver operating characteristic (ROC) curves assessing the sensitivity and specificity of clinical parameters for the diagnosis of mild and severe pterygium. Dots on the curve indicate different cut-off values. AUC: area under the curve; TPR: true positive rate; FPR: false positive rate.

Diagnostic ROC curves and AUC values were used to evaluate the diagnostic accuracy of clinical parameters for distinguishing between mild and severe pterygium. The AUC for the vessel density was 0.7, with the corresponding ROC curve demonstrating statistical significance (*p* < 0.05). Further analysis demonstrated that the ROC curves for pterygium length, area and thickness, as well as vessel length, were statistically significant (*p* < 0.05) (Figure 5). The AUC value for pterygium thickness was the highest at 0.79. Setting a cut-off value of 492.73 µm for pterygium thickness resulted in a sensitivity of 0.81 and a specificity of 0.67.

## Discussion

This study highlights that pterygium is not merely a static lesion but a progressive disorder with distinct vascular remodelling patterns. Increased vessel density, coupled with vessel dilation in mild cases and branching in severe cases, underscores the dynamic nature of angiogenesis in this condition. These observations suggest that vascular changes might have played a pivotal role in the pathogenesis and progression of pterygium, offering potential avenues for targeted interventions.

### Progressive vascular remodelling as a hallmark of pterygium pathogenesis

This study provides novel insights into the dynamic progression of pterygium through quantifiable vascular remodelling. The significant increases in vessel density (control: 53.26% vs. mild: 63.16% and severe: 69.59%, *p* < 0.001) and morphological shifts – vessel dilation in mild stages (diameter: 17.96 ± 2.12 µm) versus neovascular branching in severe cases (length: 167.50 ± 23.14 µm vs. control, *p* = 0.01). These findings align with histopathological evidence of dense microvascular networks in pterygium [19], yet extend prior work by linking specific vascular patterns to clinical severity.

### Pterygium thickness: a biomarker for grading and monitoring

Our data establish pterygium thickness (severe: 518.21 ± 89.58 µm vs. mild: 403.72 ± 105.26 µm, *p* < 0.001) as the robust diagnostic marker (AUC = 0.79). This parameter, measurable only via AS-OCT or ultrasound biomicroscopy, outperformed traditional metrics like length or area [20,21]. Notably, our measurements corroborate Raj et al.’s AS-OCT data (455.78 ± 188.34 µm) [21], while Hilmi et al.’s validation of AS-OCT superiority over slit-lamp biomicroscopy [22] underscores its clinical utility for surgical planning.

### Vascular morphology and inflammatory mechanisms

The increased vessel diameter in mild pterygium likely reflects inflammation-driven vasodilation. Turan et al.[23] found that vascular endothelial cells and inflammatory cells (mast cells) were significantly higher in primary pterygium than in normal conjunctiva. Inflammatory factors, such as interleukin-1, interleukin-6, interleukin-8 and nitric oxide (NO), are upregulated in pterygium [24]. These mediators promote endothelial NO release, inducing vasodilation. Conversely, severe pterygium showed no diameter increase but the shortest vessel length, which may be attributed to neovascular branching complexity. As new vessels proliferate, they form a dense, fragmented network, reducing average vessel length – a compensatory response to hypoxia. This highlights distinct remodelling phases: dilation/lengthening in early stages vs. branching/shortening in advanced disease.

### Sex-specific haemodynamics and age-related progression

While hemodynamic parameters (Va, Vs and *Q*) did not correlate with severity, female sex showed a positive association with flow velocities (*r* = 0.405–0.410, *p* < 0.001), potentially linked to higher NO levels in females [25–27]. NO-mediated vasodilation and reduced blood viscosity may explain this trend, though hormonal influences (e.g. oestrogen’s endothelial protective effects) require further exploration. Additionally, pterygium size correlated strongly with age (*r* = 0.441 for length, *r* = 0.373 for area, *p* < 0.001), consistent with age being a recognized risk factor [3,28] and Fernandes et al. [4] association of older age with ≥3 mm pterygium.

### Limitations and future directions

While AS-OCTA and FSLB provided vascular detail, this study still has limitations. Its cross-sectional design prevents causal inferences, and the relatively small sample size may limit statistical power for subgroup analyses, potentially explaining the lack of significant differences in certain hemodynamic parameters which may exhibit high individual variability. The lack of longitudinal data also restricts tracking of temporal vascular changes. Notably, the quantitative vessel density and thickness identified here show promise as non-invasive biomarkers for severity grading. Longitudinal studies tracking vascular changes pre/post-surgery could clarify remodelling causality. Larger, multi-centre cohorts are needed to confirm findings. Additionally, sex-specific NO modulation and oestrogen/androgen receptor profiling should be prioritized to decode observed hemodynamic trends. Furthermore, the detailed vascular characterization provided by this study lays a foundation for optimizing anti-angiogenic therapies, potentially as adjuvants to improve surgical outcomes.

In conclusion, this study reveals substantial variations in pterygium characteristics and vascular parameters, indicating the progressive nature of this condition. In terms of vascular density, pterygium conjunctiva has significantly higher vascular density compared to normal conjunctiva, with severe pterygium having even higher density than mild pterygium. This suggests that neovascularization may play a crucial role in the pathogenesis and progression of pterygium. Regarding vascular morphology, pterygium vessels undergo changes with dilation and lengthening in mild cases, and increased neovascularization and branching with shortened vessel lengths in severe cases, indicating that vascular remodelling correlates with disease severity. However, no significant hemodynamic changes were observed in pterygium. These findings contribute to a refined understanding of the vascular and hemodynamic intricacies inherent in pterygium pathophysiology, laying the groundwork for targeted therapeutic interventions and prognostic assessments.

## Data Availability

The data supporting the findings of this study are available from the corresponding author, Liang Hu, upon request.
